# Utilization of Bottom Ash from Biomass Combustion in a Thermal Power Plant to Remove Cadmium from the Aqueous Matrix

**DOI:** 10.3390/molecules29235727

**Published:** 2024-12-04

**Authors:** Eva Pertile, Tomáš Dvorský, Vojtěch Václavík, Bohdana Šimáčková, Lukáš Balcařík

**Affiliations:** Department of Environmental Engineering, Faculty of Mining and Geology, VSB—Technical University of Ostrava, 17. listopadu 15/2172, 708 00 Ostrava, Czech Republic; eva.pertile@vsb.cz (E.P.); vojtech.vaclavik@vsb.cz (V.V.);

**Keywords:** plant biomass, biomass combustion, bottom ash, adsorption, cadmium

## Abstract

This study provides a cost-effective method for using bottom ash from biomass combustion, which would otherwise constitute waste, to remove cadmium from acidic industrial wastewater. The X-ray powder diffraction method was used to identify the crystal forms, i.e., the arrangement of atoms in the crystal lattice, and to determine the composition of bottom ash, and the X-ray fluorescence method was used to obtain information on the elemental composition of bottom ash. The Fourier Transform Infrared method was used to analyse and identify the different functional groups occurring in bottom ash. Scanning Electron Microscopy with energy-dispersive X-ray was used to obtain detailed information on the bottom ash surface. The effect of various factors on Cd removal was studied, and optimal experimental conditions were found. The kinetic and thermodynamic equations showed that the removal of Cd^2+^ using bottom ash from biomass combustion was a single-layer chemical adsorption meeting the requirements of pseudo-second-order kinetics. The limiting parameter for the effective adsorption of Cd^2+^ using bottom ash from biomass combustion is its alkaline nature. It can only be used for solutions with pH < 2, which, on the other hand, is its advantage in practical application, namely, in the final treatment of acidic industrial wastewater.

## 1. Introduction

Water pollution is a global threat, not only because of water scarcity, but also because of the increasing amount of wastewater. While rapid industrial and economic growth brings progress, it also increases the level of contamination of water resources, thus raising concerns about the future. Due to worsening conditions stemming from global warming and excessive groundwater and surface water consumption, it is crucial to look for effective solutions for wastewater treatment and recycling [1].

Water pollution by heavy metals from industrial activities is increasing and becoming a global problem [2]. Mining, mineral processing and metallurgical operations produce wastewater containing heavy metals. Heavy metals in wastewater are persistent and non-degradable in the environment. They can be soluble in the aquatic environment and are easily absorbed by living organisms. As they enter the food chain, they bioaccumulate and biomagnify at higher trophic levels. Heavy metals can cause serious health problems if ingested above acceptable levels. Industrial wastewater containing heavy metals must therefore be treated before discharge [3,4]. Cadmium (Cd^2+^) is one of the best-known toxic environmental pollutants whose widespread use for industrial purposes has raised various environmental and human health concerns worldwide [5].

In water, cadmium occurs mainly as free ions (Cd^2+^) or as complexes with inorganic or organic ligands. Its speciation and mobility depend on factors such as pH, redox potential and the presence of other ions or organic matter. At lower pH, Cd is more soluble and bioavailable, which increases its toxicity [6]. Cadmium is highly toxic to aquatic organisms even at low concentrations. It interferes with enzyme activity, damages cell membranes and disrupts calcium metabolism, which can lead to impaired reproduction, growth and survival of fish and other aquatic species. In humans, exposure to cadmium through contaminated water and food can lead to serious health problems, including kidney damage, skeletal demineralisation and an increased risk of cancer [7]. The bioaccumulative nature of cadmium further exacerbates its environmental and health impacts and highlights the need for effective removal strategies from industrial and agricultural effluents to protect aquatic ecosystems and human health [8]. Due to its toxicity, Cd has been added to the list of toxic chemicals in the European Water Framework Directive [9].

Cadmium enters natural waters mainly from industrial processes such as mining, electroplating, battery production, pigment production and electronic waste disposal. In addition, agricultural activities contribute to cadmium contamination through their use of phosphate fertilisers and sewage sludge, which often contain cadmium as an impurity. Atmospheric deposition from the burning of fossil fuels can also lead to cadmium leaching into water systems. Despite the various monitoring measures taken to limit the release of Cd^2+^ into the environment, pollution levels are still alarming [10].

Various conventional methods are used to limit the release of Cd^2+^ into the environment, such as chemical precipitation, chemical oxidation or reduction, coagulation [11]), evaporation, ion exchange [12], reverse osmosis, electrochemistry, solvent extraction [13], membrane filtration [14], cementation and activated carbon adsorption [15,16]. However, conventional technologies produce a large amount of sludge, which is difficult to dispose of and acts as a secondary pollutant. It is therefore important to develop an economical technique for the removal of Cd^2+^ from wastewater.

Due to increasing environmental pollution, there is currently a growing demand for a variety of new low-cost, high-efficiency adsorbents for wastewater treatment [17,18]. Unconventional and inexpensive adsorbents, such as natural materials or agricultural and industrial wastes, can be effectively used for the adsorptive removal of metal ions from industrial and municipal wastewater. Adsorption is a rapid and versatile method due to its cost effectiveness, ease of operation and efficient implementation [19]. There has been considerable research into adsorption technology for the removal of cadmium ions from aqueous solutions [20]. Activated carbon (AC), as the most used adsorbent, has a good adsorption performance; however, its large-scale use is limited by process difficulties such as the dispersion of activated carbon powder and the high cost of regeneration [21].

Nowadays, the great demand for highly porous nanostructures such as graphene, fullerenes, graphitic carbon nitride and metal–organic frameworks (MOFs) have also been discussed as alternatives to remove heavy metals from the contaminated water, considering the advantages of nanotechnology. Therefore, such nanomodified adsorbents may offer opportunities to provide future solutions to the problem of water pollution [22].

With the growing global initiative to minimize waste, and with respect to European measures that aim to reduce landfilling by 2030, new ways to efficiently use the by-products of industrial processes are being explored. One such option is the use of ash from biomass thermal power plants as a potential adsorbent of heavy metals from industrial wastewater. This approach not only reduces the concentration of harmful substances in the water, but also contributes to the circular economy as new uses are found for the ash.

The use of ash from biomass combustion to remove toxic metals from wastewater has a great potential. The ability of ash to bind metals depends on its physical and chemical properties and its composition. Ash contains porous and amorphous particles of different sizes that can adsorb metals on their surface [23]. Biomass combustion ash is suitable for this purpose due to its higher contents of calcium oxide (CaO) and magnesium oxide (MgO) [24]. The main objective of this research was to assess whether the bottom ash from biomass combustion can be effectively used to reduce cadmium (Cd) concentrations from an aquatic environment.

## 2. Results and Discussion

### 2.1. Physical–Chemical Properties of Bottom Ash

Since the adsorbent used can have a major influence on the course of metal adsorption, attention was first paid to its detailed study. The ash originating from the combustion of plant biomass can contain a wide range of elements, and their abundance will vary depending on the type of plant biomass burnt, on the extent of contamination of the soil pool with toxic metals where the biomass grew, on the use of unsuitable or prohibited fertilisers and, last but not least, on the type of ash. The results of the elemental analysis obtained by the XRF method are presented in Table 1. The amount of elements present in the bottom ash from the combustion of plant biomass is about 85% *w*/*w* (XRF analysis cannot analyse carbon, hydrogen, nitrogen and oxygen). Elements with a content higher than 20% *w*/*w* are shown in red, and elements present in the ash in the amount of around 1–2% *w*/*w* are shown in blue.

The values presented in Table 1 show that the bottom ash from the combustion of plant biomass is not contaminated with toxic metals. It can be concluded that the used bottom ash has contents of Si (approx. 29% *w*/*w*), K (approx. 25% *w*/*w*) and Ca (approx. 20% *w*/*w*). P and S are present in the amount of 2% *w*/*w*.

**Figure 1:**
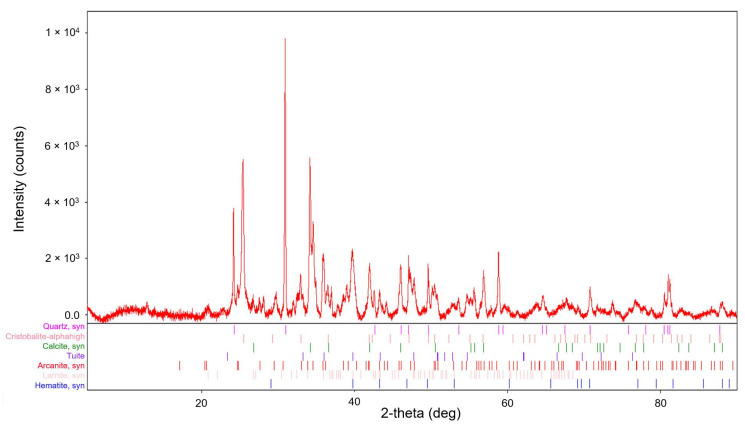
Diffractogram and phase composition of bed ash from combustion of plant biomass (range 5–90° 2theta).

The X-ray powder diffraction technique was used to study the crystal structure of the bottom ash. The diffractogram and phase composition of bed ash is shown in Figure 1.

**Figure 2:**
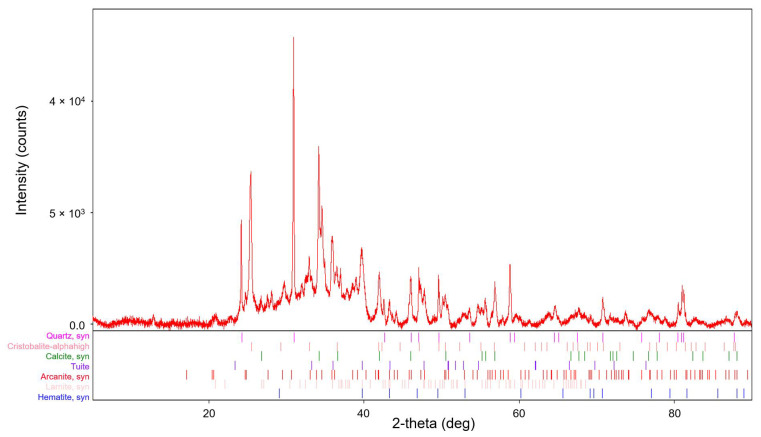
Diffractogram and phase composition of the fly ash from the combustion of plant biomass (range 5–90° 2theta).

The amorphous carbon is very visible in the XRD spectrum of the fly ash. It is a “hump” in the region around 30 degrees 2θ [25]. The diffractogram obtained and the phase composition of the fly ash are shown in Figure 2.

The measured sample was compared with the International Centre for Diffraction Data (ICDD) reference diffractogram database PDF-2. The resulting phase composition of the sample, including the PDF-2 reference diffractogram number, is presented in Table 2.

Polymorphic minerals containing SiO_2_ (silica and cristobalite) were present in the bottom ash from the combustion of plant biomass. Cristobalite is essentially a high-temperature form of silica (SiO_2_). Other typical minerals formed at high temperatures are larnite or haematite. Calcite was also present and is likely to be a product of the combustion of plant biomass containing calcium compounds. Rarer minerals, such as tuite or arcanite, were found here as well. The crystal composition thus confirmed the XRF analysis in terms of its elemental composition.

**Figure 3:**
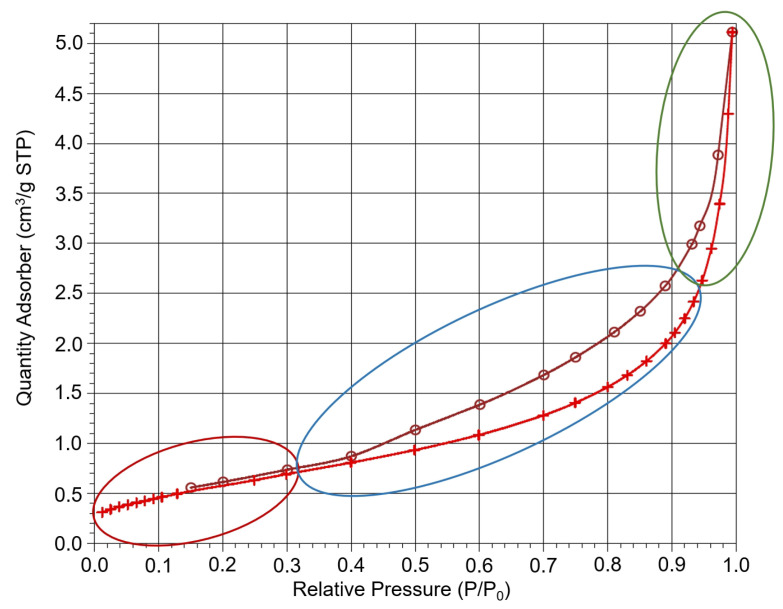
Adsorption and desorption isotherms of bottom ash. Mesopores area—blue ellipse, macropores area—green ellipse, micropores area—red ellipse. Red line with crosses—adsorption curve, dark red line with circles—desorption curve.

During the adsorption, the ability of the adsorbent to trap dissolved metals on its surface is exploited. The specific surface area is thus one of the basic characteristics of each adsorbent. Based on the physisorption results, the specific surface area of the bottom ash from the combustion of plant biomass was found to be only 2.22 m^2^ g^−1^. Compared to the surface area of activated carbon (>400 m^2^ g^−1^), which is one of the most efficient adsorbents, the specific surface area of the studied adsorbent is extremely low. This fact was also confirmed by calculation, where the total pore volume of V_net_ was 7.9081 mm^3^ _lig_ g^−1^. The total pore volume corresponds to the surface area of SBET 2.2186 ± 0.0184 m^2^ g^−1^. Based on the linear growth of curve in case of the relative pressure of 0–0.3 p/p_0_ (see Figure 3), it can be concluded that the studied adsorbent is minimally microporous or is not microporous at all.

**Figure 4:**
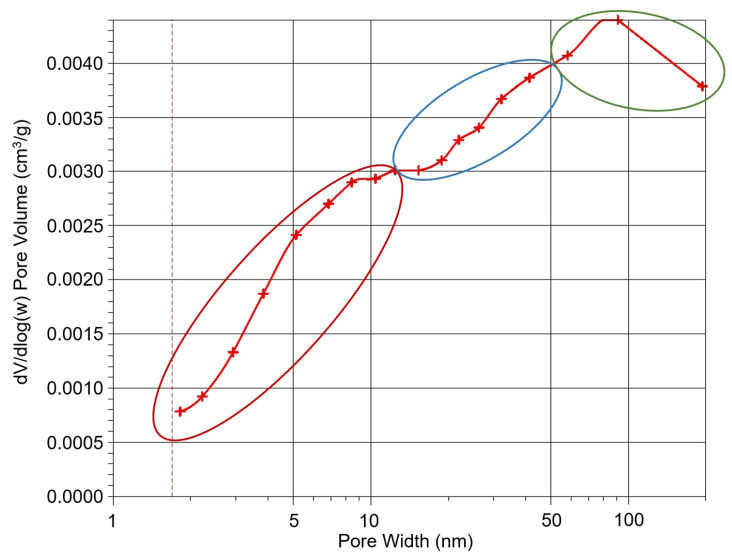
Pore size of the adsorbent calculated according to the BJH model, taking into account the Carbo black STSA model (cylindrical pore volume). Note to Figure 4: Values on the *x*-axis are logarithmized for clarity. Mesopores area—blue ellipse, macropores area—green ellipse, micropores area—red ellipse.

Mesopores are the most abundant in the bottom ash sample (marked in blue). The green part represents the macroporous area. It can be therefore concluded that bottom ash from the combustion of plant biomass can be considered as a meso–macroporous adsorbent. This is also confirmed by the pore distribution, which is graphically shown in Figure 4, where most of the pores are in the macroscale area, i.e., within the area of 70–80 nm.

The results obtained from SEM analysis were found to be in agreement with those obtained from physisorption. Therefore, based on the physisorption data, it can be concluded that the material is meso/macro porous. The curve shown in Figure 3 and Figure 4 illustrates that the material contains a small proportion of micropores, while the majority of the pores are meso- and macropores.

The observation that biomass ash exhibits good adsorption performance despite having a meso or macroporous structure and a low specific surface area can be explained by considering several factors. Although the specific surface area is low, the presence of meso- and macropores may facilitate the diffusion of larger molecules into the ash structure, thereby increasing the adsorption capacity for certain types of adsorbates [26]. The chemical composition and surface functional groups of biomass ash, such as hydroxyl, carboxyl and other oxygen-containing groups, can interact with adsorbates through hydrogen bonding, electrostatic interactions and van der Waals forces [27]. In addition, biomass ash may contain minerals and metal oxides that can participate in ion exchange processes and contribute to the adsorption of ionic species from solutions [28]. The macroporous structure can physically trap larger particles or molecules, which can contribute to the overall adsorption capacity [29]. In addition, the heterogeneous nature of biomass ash surfaces can provide a variety of adsorption sites, each with different affinities for adsorbates, thereby enhancing the overall adsorption performance [30]. The observation that biomass ash has good adsorption performance despite having a meso- or macroporous structure and a low specific surface area can be explained by considering several factors [31].

**Figure 5:**
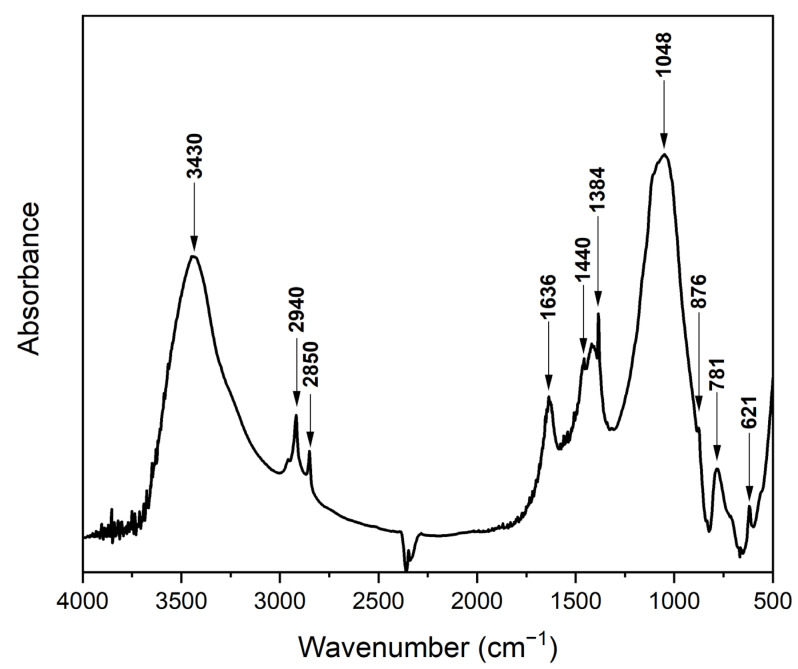
FTIR spectrum of bottom ash from the combustion of plant biomass in a thermal power plant.

The chemical properties of the adsorbent used can also significantly affect the course of metal adsorption. FTIR spectra were used to analyse the surface layers of the bottom ash (Figure 5).

The band ranging from 3700–3000 cm^−1^ is clearly visible from the FTIR spectrum. This is usually attributed to the hydroxyl (-OH) functional group, which is typical for humidity. However, this humidity is typical for analysis using KBr tablets and is therefore attributed to the hydroscopic properties of KBr. The peak with a wavenumber of 1630 cm^−1^ is not typical for the hydroxyl group but directly for the free water contained in the sample. In the rest of the FTIR spectrum, it is visible that the sample was very well carbonized during the process of the combustion of plant biomass, and thus outweighs the CH_2_ functional groups of asymmetric (2940 cm^−1^) and symmetric (2850 cm^−1^) vibrations. C-H groups are also present here, and they are attributed to peaks with wavenumbers of 1440 cm^−1^, 1380 cm^−1^, 876 cm^−1^ and 781 cm^−1^. A very interesting functional group occurs in the spectrum at a wavenumber of 1048 cm^−1^, based on peak deconvolution using Omnic software (OMNIC 9.14.97); it could be a C-O-H group, but it could also be a C-O, C-C or C-H group [32]. The formation of primary alcohols and their groups is due to the process of thermal decomposition of carboxylic acids contained in plant biomass. At the end of the spectrum, there is the SO_4_^2−^ group (621 cm^−1^), which corresponds to an ash composition of 2.282 wt.% S [33].

**Figure 6:**
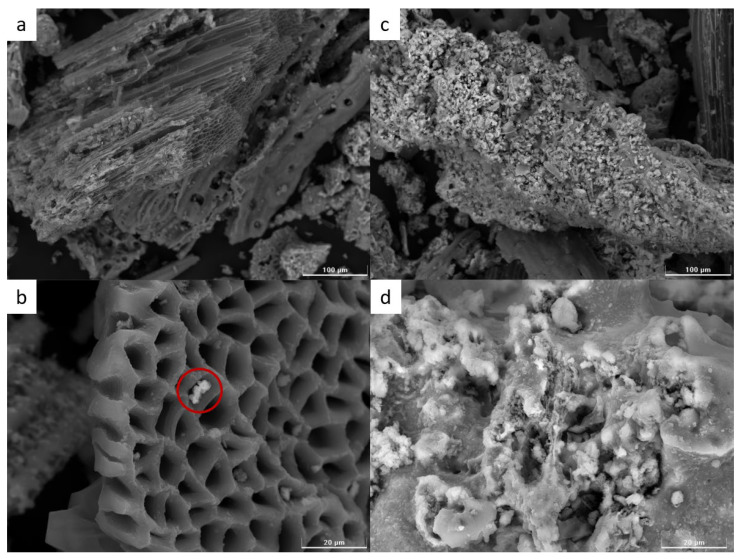
Ash surface morphology. (**a**) Remains of the plant biomass; (**b**) zoomed remains of the plant biomass, the red circle indicating the metal in the porous material structure; (**c**) crystallinity on the surface of the material; (**d**) zoomed image of crystallinity on the surface of the material.

SEM was used to take images of the surface of the sample, which were used to determine the exact surface morphology. Based on the results obtained from the physisorption, it can be concluded that the surface of the adsorbent used is mainly meso–macroporous. This is also confirmed by the photographs in Figure 6, which show that the surface of the bottom ash is not uniform. Figure 6a clearly shows the remains of the plant biomass. Volatile substances are released first during the combustion process, followed by the combustion itself. If the amount of oxygen is less than the stoichiometric coefficient, only plant biomass is carbonized. The formation of porous material is the side effect of the charring process, as can be seen in the closer view in Figure 6b, where clearly porous material is visible. Different shades of grey in the red circle are noticeable as well. One part is brighter here because the metal represented here emits electrons, and thus appears significantly brighter in the photograph than only carbonaceous material. The same effect can be seen between Figure 6c,d. This is only the zoomed image again. However, in Figure 6d, there is some kind of crystallinity on the surface of the material, as well as amorphousness.

The amorphous designation, then, belongs to the entire cemented surface. In the centre of photo 6d, it can be seen that the material in the centre will be porous, but due to the sintering of the surface with metal/semi-metal (Ca, K, etc.), the pores are closed.

### 2.2. Evaluation of Cd Adsorption Kinetics

The study of the kinetics of Cd adsorption focused on bottom ash from the combustion of plant biomass is important for understanding the rate of the adsorption process and, ultimately, for optimizing the experimental conditions necessary to achieve its maximum efficiency. The kinetics of metal adsorption can be described by various mathematical models that include rate constants and parameters, such as diffusion of the metal onto the adsorbent surface and internal diffusion within the porous structure of the adsorbent. The particle size of the adsorbent used plays an important role, not only in the actual adsorption process, but also in determining the costs associated with its treatment for practical applications. Smaller particles may be suitable for rapid removal of metal from a solution, but larger particles may, on the other hand, be more suitable for longer adsorption processes where a lower amount of reuse of the adsorbent is desired.

**Figure 7:**
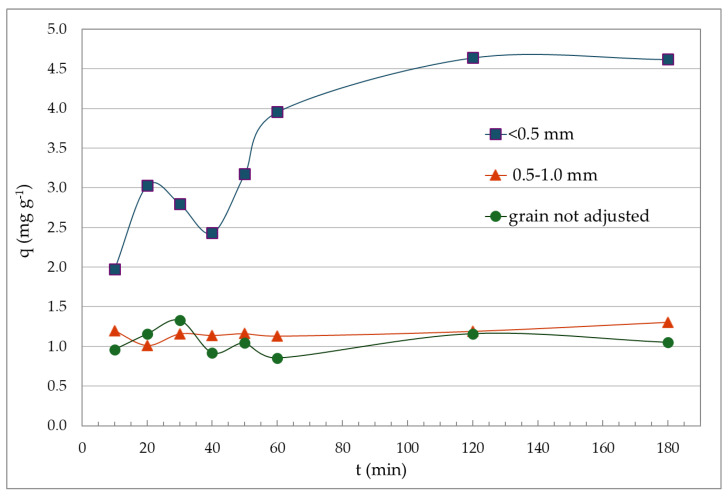
Impact of the particle size of the bottom ash depending on the contact time between adsorbent and adsorbate (Cd) (Experimental conditions: c_s_ = 20 g·L^−1^; c_i_ = 100 mg·L^−1^; 180 rpm; t = 23 ± 1 °C).

Mass transfer is provided in relation to the particle size, and it may affect adsorption efficiency to some extent. In order to facilitate grain-size sorting and also because particles of smaller size might be poorly wetted by the adsorbate (slower sedimentation properties), the whole fraction < 0.5 mm was used. A vast majority of authors also agree on the higher adsorption capacity when using fine-grained material. In general, smaller particles should be preferred for the adsorption processes, as this will provide a larger active surface area, thus making more binding sites available. This fact is verified by several authors [34,35,36,37]. On the other hand, according to Naja and Volesky (2011), particles with larger spherical shapes may contribute to higher metal adsorption, which they attribute to the contribution of external (surface) diffusion and subsequent mass transport inside the sorbent particles [38]. To remove Cd from the model solution, bottom ash of different grain size fractions was used to study the adsorption kinetics. Based on the results of sieve analysis, it can be concluded that fly ash derived from the combustion of plant biomass predominantly consisted of particles with grain sizes ranging from 0.315 to 0.5 mm (36 wt.%) and 0.180 to 0.315 mm (31 wt.%). In contrast, the fraction exceeding 3.0 mm represented the smallest proportion, accounting for only 1 wt.%, primarily comprising cinder fragments and plant debris. Particles smaller than 0.5 mm, which were utilized in the experiments, constituted approximately 86 wt.% of the sample. The effect of adsorbent particle size on Cd adsorption at different contact times (10, 20, 30, 40, 50, 60, 120 and 180 min) is evaluated graphically in Figure 7.

The graphical evaluation of the effect of particle size on the efficiency of bottom ash to adsorb Cd as a function of the contact time of adsorbent and adsorbate (Figure 5) clearly shows that the adsorption efficiency of bottom ash with a grain size of 0.5–1.0 mm and untreated adsorbent is low. However, what is more interesting is the course of the adsorption curve for the adsorbent with a grain size fraction < 0.5 mm, where 95% of cadmium was removed after 120 min (q_120_ = 4.64 mg g^−1^).

Two kinetic models were used to clarify the mechanism of Cd adsorption, as well as its potential rate-controlling steps involving mass transport and chemical reactions, and to analyse the experimental data: pseudo-first-order model and pseudo-second-order model. The kinetic parameters found for both models are shown in Table 3. The validity of the models was evaluated by determining the correlation coefficient (R^2^), where R^2^ values > 0.950 were considered satisfactory in order to describe the studied adsorbent.

The pseudo-first-order model is suitable for homogeneous surfaces and physical adsorption. When used, it was found that the values of the correlation coefficients for Cd are very low, and the model is therefore not suitable for describing the experimental data.

The data obtained from the study of Cd adsorption kinetics can be described using a pseudo-second-order model for all grain-size forms. Based on this fact, it can be concluded that the adsorption process probably depends more on the chemical interaction between the adsorbent and the adsorbate. This model is associated with chemisorption mechanisms where electron sharing, or exchange between the adsorbent and adsorbate, occurs. It assumes that the rate of adsorption is proportional to the square of the number of vacancies on the adsorbent surface, often reflecting more complex interactions such as chemical bonding. The pseudo-second-order rate constant k_2_ expresses the rate of reaction. Its lower values (around 0.001 to 0.01 g mg^−1^ min^−1^) are associated with slower adsorption or diffusion limitation [39,40]. The value of the k_2_ constant for cadmium is within the range of 0.129–0.592 g mg^−1^ min^−1^. This points to a faster adsorption process and good interaction between adsorbent and adsorbate or to high availability of active sites on the adsorbent surface.

The value of the adsorption initiation rate h_2_ provides information about the initial adsorption rate at time t = 0. In the case of cadmium, h_2_ values range from 0.55 to 1.86 mg g^−1^ min^−1^. Although the higher value of h_2_ for the grain fraction of 0.5–1.0 mm means that the adsorption of Cd has a faster onset in the initial phases, the adsorbent efficiency is very low, considering the low value of the equilibrium adsorption capacity (q_120_ = 1.51 mg g^−1^).

### 2.3. The Impact of pH Value on the Adsorption of Cd

**Figure 8:**
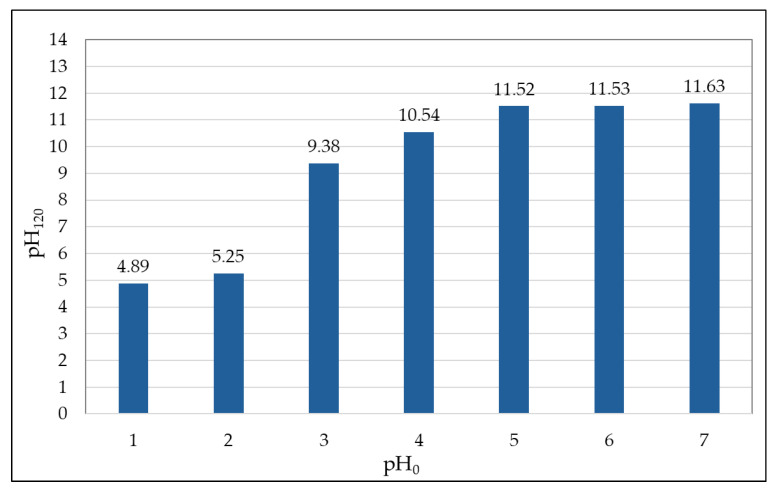
Change in the pH value of the Cd model solution after 120 min of contact time between adsorbent and adsorbate (Experimental conditions: c_s_ = 20 g·L^−1^; c_i_ = 100 mg·L^−1^; 180 rpm; t = 23 ± 1 °C, contact time 120 min, grain-size fraction < 0.5 mm).

The pH value is of great importance in the study of adsorption and can have a significant effect on the overall course of the removal of metals from the aqueous environment. During the experiments carried out in the kinetics study, it was found that, with respect to the alkaline properties of the adsorbent, mainly in the one with a lower grain-size fraction (<0.5 mm), the pH value increased with increasing contact time between the adsorbent and the adsorbate. Considering its significant influence, it was first verified for the selected bottom ash of a grain-size fraction <0.5 mm how this parameter would vary depending on the contact time (120 min). That is why the model solutions of the tested metal were prepared with different pH values ranging from 1.0–7.0. The pH value was measured at the beginning of the adsorption process (pH_0_) and also in the filtrate after the end of the adsorption process, i.e., after 120 min (pH_120_). Graphically, the change in the pH value of the cadmium model solution after 120 min of contact time between the adsorbent and the adsorbate is presented in Figure 8.

With increasing contact time of the adsorbent with the adsorbate, the pH value increases (after about 10–20 min). From the graph showing the change in pH value of the model Cd solutions after a contact time of 120 min (selected based on kinetics study), it is very clear that after two hours of contact, the pH value increases, which means that the effect of pH value on the adsorbent efficiency cannot be objectively evaluated. Most likely, the metal under study will already be present in the solution in its sparingly soluble hydroxides, so it will not be a mere adsorption, but a precipitation process will already be involved.

For further study of the mechanism of Cd adsorption using bottom ash, the following parameters were chosen based on the results: grain-size fraction < 0.5 mm and contact time of 2 h (q_120_ = 4.64 mg·g^−1^). Considering the non-negligible influence of the adsorbent on the resulting pH value, where it significantly increased towards alkaline values, a limiting value of pH = 2.0 was set for the equilibrium Cd-adsorption studies. Based on these conditions, the pH was monitored throughout the equilibrium study of the adsorption process.

The use of a buffer could be a possible solution in order to keep the pH of the solution constant throughout the entire adsorption. However, its use would be limited to a type of buffer that would not interfere with the adsorption process by its composition. Another possible solution could be to wash the bottom ash with water or a weak acidic solution, but from a practical and economic point of view, and in relation to a sustainable adsorption process, this treatment procedure would be unsatisfactory.

### 2.4. Evaluation of Adsorption Isotherms

The comparison of the adsorption capacity of single-metal adsorption can be carried out based on the adsorption isotherm for a single metal. Two models for adsorption isotherms were used to describe the data obtained in the equilibrium study: the Langmuir and Freundlich models. Both models have been extensively used by other authors who have studied the equilibrium between a metal solution and the solid phase during adsorption [38,41,42]. Although the Langmuir model does not provide any clarification of the adsorption mechanism, it can inform about the metal-uptake capacity of the studied adsorbent, and it can reflect the behaviour of the equilibrium adsorption process. The most commonly used Langmuir model is the one which contains two very useful and easy-to-understand parameters (q_max_ and K_L_) that reflect two important characteristics of the adsorption system. However, when applying it to molecular types (adsorbents), it must be considered that the accepted assumptions of these original relationships essentially come from experiments that have been carried out with activated carbon used as the solid adsorbent. The value of the maximum adsorption capacity q_max_ can also be explained as the total number of binding sites available for adsorption, and the adsorption capacity at the best exposure time q_t_ is the number of binding sites occupied by the adsorbate at a given input concentration.

Equilibrium data were obtained by changing the initial concentration of the metal under study within the concentration range of 100–1000 mg L^−1^, while the other parameters that were chosen based on the kinetics study were retained. The values of the maximum adsorption capacity q_max_ and other constants of the two isotherm models, including the individual correlation coefficients R^2^ obtained from the evaluation of the adsorption isotherms for cadmium, are presented in Table 4.

**Figure 9:**
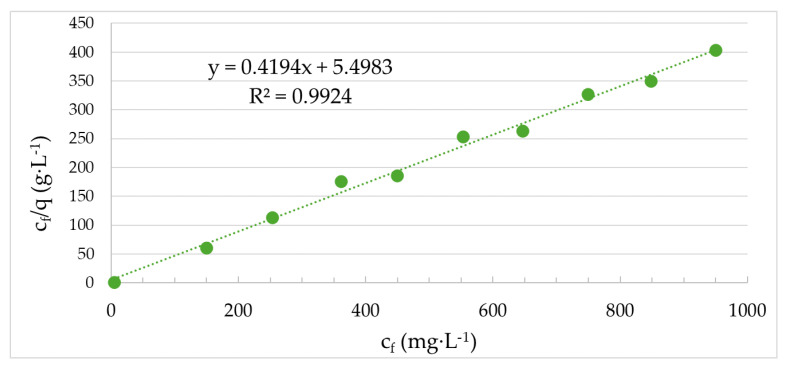
Linear Langmuir isotherm of Cd adsorption using bottom ash (Experimental conditions: c_s_ = 20 g·L^−1^; 180 rpm; t = 23 ± 1 °C, without pH adjustment, contact time of 120 min, grain-size fraction <0.5 mm).

The Langmuir adsorption isotherm, which is suitable for the description of chemisorption and electrostatic adsorption, where only one layer of adsorbate is formed and all active sites on the surface are equal, can be used to describe the adsorption data in the case of bottom ash from plant biomass combustion (Figure 9). It can therefore be concluded that adsorption takes place on a specific adsorbent surface, where each adsorption site can be occupied by only one adsorbate molecule.

The linearized Langmuir model was used to calculate the maximum adsorption capacity of cadmium (*q_max_* = 2.38 mg·g^−1^), which corresponds to the experimental value of adsorption capacity (*q_120_* = 2.35 mg·g^−1^). The initial slope is another important characteristic of the adsorption isotherm curve. A steep initial slope indicates that the adsorbent will have a good adsorption capacity for adsorbate within the low residual-concentration range c_f_. This affinity of the adsorbent for the adsorbate is indicated by the Langmuir constant K_L_. The lower the K_L_ value, the higher the affinity of the adsorbent for the adsorbate should be. Considering the very low value of the Langmuir constant K_L_ (0.021 L·mg^−1^), it could be assumed that the affinity of the bottom ash to cadmium is favourable. In general, however, for “good” adsorbents, it is necessary to find not only a steep slope of the initial sorption isotherm, i.e., a low value of the Langmuir parameter K_L_, but also a high value of *q_max_* [43]. Taking into account the low calculated value of the adsorption equilibrium parameter R_L_ (R_L_ = 0.048 for an input Cd concentration of 1000 mg·L^−1^), the adsorption of cadmium using bottom ash will have a favourable course because its value is between 0 and 1. It was also found that the bottom ash from the combustion of plant biomass will have higher adsorption efficiency, especially for lower cadmium concentrations, so it can be applied in the treatment of industrial wastewater before its discharge to the sewer system. Bottom ash from the combustion of plant biomass, although with half the efficiency, can adsorb cadmium over a relatively wide concentration range (from 200 mg L^−1^ to 1000 mg·L^−1^). Based on the facts presented above, untreated bottom ash from the combustion of plant biomass could only be applied to the final treatment of industrial wastewater containing residual cadmium concentrations.

**Figure 10:**
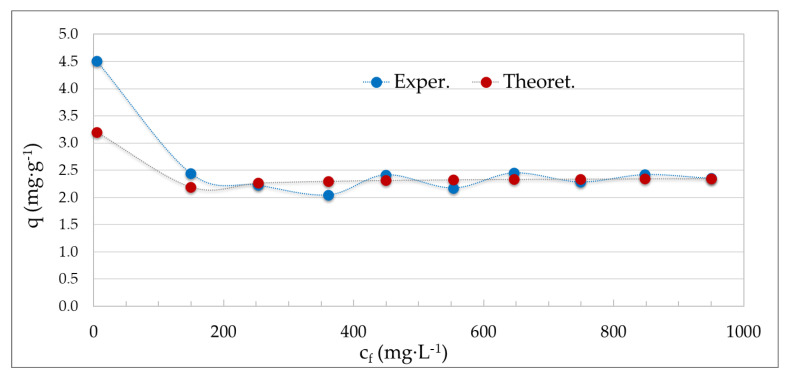
Non-linear Langmuir isotherm of Cd adsorption using bottom ash (Experimental conditions: c_s_ = 20 g·L^−1^; 180 rpm; t = 23 ± 1 °C, contact time 120 min).

From the linear dependence of c_f_/q on c_f_ of the Langmuir equilibrium model, the constants necessary for the calculation of the non-linear dependence of the equilibrium sorption capacity *q* on the equilibrium concentration of adsorbate in solution c_f_ were found. It indicates the agreement of the experimentally determined sorption capacities with the theoretical ones. The following Figure 10 shows the non-linear Langmuir isotherm for the adsorption of cadmium using bottom ash from the combustion of plant biomass.

The non-linear dependence shows that the curves for both experimental data (blue curve) and theoretical data (red curve) have a similar shape. From the course of both curves, the bottom ash from the combustion of plant biomass will have a higher adsorption efficiency, especially for lower cadmium concentrations, which goes down to a half in case of higher concentrations. It is positive, however, that it can adsorb cadmium over a relatively wide concentration range, i.e., from 100 mg·L^−1^ to almost 1000 mg·L^−1^.

The composition of bottom ash includes significant amounts of crystalline SiO_2_, CaCO_3_ and Ca_3_(PO_4_)_2_, among other compounds, which can influence the process of cadmium (Cd) adsorption. Silica (SiO_2_) provides surface areas that facilitate Cd sorption through physical adsorption and chemical bonding, with surface hydroxyl groups interacting with Cd^2+^ ions to enhance sorption efficiency [44]. Calcium carbonate (CaCO_3_) can impact cadmium sorption primarily through ion exchange and complex formation, where cadmium ions react with carbonate ions to form insoluble precipitates, reducing cadmium’s mobility and bioavailability [45]. Similarly, calcium phosphate can contribute to cadmium immobilization by forming insoluble phosphate complexes, effectively limiting the availability of cadmium in a solution [46]. These interactions underline the potential of bottom ash as an effective material for mitigating cadmium contamination in environmental applications.

## 3. Materials and Methods

### 3.1. Adsorbent Preparation Methodology

The adsorption experiment used bottom ash, which came from a thermal power plant that uses biomass as the renewable energy source, as the potential adsorbent. Fluid technology was used for its combustion. At the time of sampling, mixed wood chips were burned.

In order to ensure more suitable binding interactions and to improve the functionality and applicability of the adsorbent in the adsorption process (removal of the sticky effect), the samples were dried in the ECOCELL standard drying oven (BMT Medical Technology s.r.o., Brno, Czech Republic) to a constant weight at a temperature of 105 ± 1 °C.

As it was also necessary to find out the necessary information about the structure, elemental composition, and surface of the bottom ash, the samples were grain-size adjusted using RETSCH stainless steel sieves (RETSCH GmbH, Haan, Germany) to the following fraction sizes: <0.5 mm; 0.5–1.0 mm and >1.00 mm. The adsorbent was stored in a desiccator to ensure correct weighing for each experiment.

### 3.2. Methodologies Used for the Characterisation of Bottom Ash

The X-ray powder diffraction (XRD) method was used to identify the crystalline forms, i.e., the arrangement of atoms in the crystal lattice, and to determine the composition of the bottom ash. This method is based on the diffraction of X-rays on the atoms in the crystal lattice of the material, which produces characteristic diffraction images. Diffractograms were obtained using a Rigaku SmartLab diffractometer (Rigaku, Tokyo, Japan) with a D/teX Ultra 250 detector. The X-ray source was a Co lamp (CoKα, λ_1_ = 0.178892 nm, λ_2_ = 0.179278 nm) with a voltage of 40 kV and a current of 40 mA. The incident and diffracted optics were equipped with 5° Soller slits. The sample was rotated (30 rpm) during the measurements to suppress the preferential orientation effect. The measurement range was 2–90° 2theta with a step of 0.01° and a speed of 0.5° min^−1^. The measured record was evaluated using the PDXL2 software (version 2.4.2.0) and compared with the PDF-2 database released by the International Centre for Diffraction Data in 2015.

The XRF (X-ray fluorescence) method was used to obtain information on the elemental composition of the bottom ash. The principle of the method is to analyse the fluorescence radiation emitted when the sample interacts with more energy-rich X-rays. The chemical composition of the sample was determined semi-quantitatively using an X-ray fluorescence energy-dispersive spectrometer XEPOS (Spectro, Kleve, Germany). The error of determination of the XRF method expressed as relative standard deviation (RSD) is 15%.

The Fourier Transform Infrared (FTIR) method was used for the analysis and identification of the different functional groups present in the bottom ash. The principle of the method is the ability of molecules to absorb infrared radiation at specific wavelengths, which is characteristic of their chemical bonds and structure. The analysis was performed on a Thermo Scientific Nicolet iS10 FTIR spectrometer. Measurements were performed within the range of 500–4000 cm^−1^ with a resolution of 2 cm^−1^ using a KBr tablet. KBr was mixed with the sample in a 20: 1 ratio and then pressed. Omnic software was used to evaluate the ash sample along with the supplied libraries. The spectra were further evaluated based on peer-reviewed publications [33,47]. The error of determination of the FTIR-KBr method expressed as relative standard deviation (RSD) is <5%.

Scanning Electron Microscopy with energy-dispersive X-ray (SEM/EDX) Tescan Vega (Tescan group, a.s., Brno, Czech Republic) was used to obtain detailed information about the bottom ash surface. The nitrogen physisorption method was used to determine the surface and pore volume of the bottom ash. This method is used to determine the solid surface area and pore volumes. The analysis is based on the measurement of the volume of adsorbed gas depending on its pressure at constant temperature. Nitrogen physisorption was carried out using a 3Flex apparatus (Micromeritics, Norcross, GA, USA). Nitrogen adsorption–desorption isotherms at 77 K of all samples were measured for a range of relative pressures of p/p_0_ ∼1 × 10^−7^–0.99. The obtained nitrogen adsorption–desorption data were processed using BET theory, the t-plot method and the BJH method applied to the adsorption branch of the nitrogen adsorption–desorption isotherm using the standard Carbon Black STSA isotherm and assuming a cylindrical pore geometry aimed at determining the specific surface area, S_BET_ (m^2^ g^−1^), mesopore surface area, S_meso_ (m^2^ g^−1^), and mesopore size distribution.

### 3.3. Methodology for Adsorption Experiments

In order to evaluate the adsorption, with respect to its practical application and the possibility of mutual comparison of adsorbent efficiency for the studied metals, the achievement of the adsorption equilibrium state was described using unified mathematical kinetic and equilibrium models. All the adsorption studies were, for the sake of simplicity, performed in batch mode. The rate and equilibrium data of the adsorption process were obtained in this way. Analytical precision scales DENVER SUMMIT balance (Instrument Company, New York, NY, USA) with a sensitivity of 0.000 1 mg were used to weigh the samples used in all the experiments (particle size effect, pH effect, kinetics and equilibrium studies).

#### 3.3.1. Methodology for Modelling the Adsorption Process

Studying the kinetics of the adsorption process allows for a better understanding of its mechanisms, and it also makes it possible to determine the rate of the process, including the time at which the equilibrium between the two phases (adsorbent–adsorbate) is established. The best results obtained from these experiments were then used to study the equilibrium model. The kinetics of adsorption can also be influenced by multiple factors, such as the type of adsorbate and adsorbent, temperature, pressure, pH, concentration and surface properties of the adsorbent. Since bottom ash is a heterogeneous material, the kinetics study was carried out for three different types of samples (untreated fraction; 0.5–1.0 mm and <0.5 mm grain fractions).

To study the kinetics of adsorption, it was first necessary to prepare stock-model solutions of the studied metal (Cd). The input concentration that was most suitable for the dry adsorbent loading was chosen for the experiments. All adsorption experiments were performed by mixing 1.0000 ± 0.01 g of adsorbent dried to constant weight with 50.0 mL of solution of the studied metal with the concentration of 100 mg·L^−1^. The experiments were carried out in high-density polyethylene (HDPE) plastic sample containers with the volume of 100 mL. The temperature during the experiment was kept constant at 23 ± 1 °C (controlled by a temperature sensor). The samples were shaken in a shaking incubator type GFL 3031 (Helago-CZ, s.r.o., Brno, Czech Republic) at 180 rpm. min^−1^. When the predetermined exposure time (10, 20, 30, 40, 50, 60, 120 and 180 min) expired, the suspension was filtered through a PRAGOPOR 6 membrane filter (Pragochema spol. s r.o., Prague, Czech Republic), which was was used for all filtrations. The described procedure was applied equally for all the selected grain fractions.

The following relation (see Equation (1)) was used to calculate the amount of adsorbate adsorbed when establishing the equilibrium per unit of adsorbent mass, i.e., the equilibrium adsorption capacity of the adsorbent *q_r_* [39]:

(1) $$q_{r}=\frac{V\left(c_{i}-c_{f}\right)}{m}$$

where:

*q_r_*: equilibrium adsorption capacity of the adsorbent, (mg·g^−1^),

*c_i_*: real initial concentration of adsorbate in solution, (mg·L^−1^),

*c_f_*: equilibrium concentration of adsorbate in solution, (mg·L^−1^),

*m*: mass of sorbent, (g)

*V*: volume of adsorbate solution (L).

To explain the mechanism of adsorption and its possible rate-controlling steps involving mass transport and chemical reactions, two kinetic models were used to analyse the experimental data: pseudo-first-order model (Equation (2)) and pseudo-second-order model (Equation (3)) [48,49]:

(2) $$log\;\left(q-q_{t}\right)=\log q-\left(\frac{k_{1}}{2.303}\right)t$$

(3) $$\frac{t}{q_{t}}=\frac{1}{k_{2}q^{2}}+\frac{t}{q}$$

Mass k_2_ was obtained from the dependence of *t/q_t_* on time *t,* and it was used to calculate the initial adsorption rate *h* (mg g^−1^ min^−1^), v t→0. See Equation (4) [50]:

(4) $$h=k_{2}q^{2}$$

#### 3.3.2. Methodology Used to Study the Impact of pH

The impact of pH value on the adsorption process of Cd was verified only for bottom ash of the grain size that showed the highest removal efficiency of the metal in question within the scope of the kinetics study. The study was carried out for pH values between 1.0–7.0. Higher pH values were not studied due to the fact that metal precipitation was already occurring. The individual pH values of the model metal solutions were adjusted using the addition of nitric acid (HNO_3_) with a concentration of 0.1 mol L^−1^ or sodium hydroxide (NaOH) with a concentration of 0.1 mol L^−1^. All the chemicals used were of analytical grade (p.a.). The actual pH value of the model metal solution was verified each time using a pH meter PH-METR 526 (WTWcz). The pH value was also measured in the filtrate. The other experimental conditions remained constant, as they were during the kinetics study. The adsorbent–adsorbate exposure time was 120 min, which was the time when the equilibrium state of the system was reached.

### 3.4. Methodology Used to Study Equilibrium

Equilibrium adsorption isotherms describe the relationship between the concentration of the adsorbed substance and the concentration of this substance in the liquid phase at equilibrium state. That is why the following experimental conditions were maintained to model the adsorption isotherms: the grain-size fraction was chosen based on the study of kinetics, where the adsorbent reached the highest removal efficiency of the metal in its equilibrium state; temperature 23 ± 1 °C; stirring speed of 180 rpm^−1^. A series of model solutions of the metal of interest were prepared with different initial solution concentrations (100, 200, 300, 400, 500, 600, 700, 900 and 1000 mg·L^−1^) to study the adsorption isotherms. All the model solutions were prepared by dilution from a stock-model solution of the metal at the concentration of 1 g L^−1^. The Langmuir and Freundlich models were used to describe the adsorption isotherms in both linearized and non-linearized forms.

The Freundlich equation in non-linear form (Equation (5)) and in linear form (Equation (6)) is as follows [39]:

(5) $$q_{e}=K_{F}c_{e}^{1/n}$$

where:

*K_F_*: the Freundlich constant, also known as the Freundlich capacity, (mg·g^−1^),

1/*n*: Freundlich constant, indicating the intensity of adsorption,

*q_e_*: the adsorption capacity, the equilibrium amount of dissolved metal adsorbed per weight unit of adsorbent, (mg·g^−1^),

*c_e_*: the equilibrium concentration of the solute in the solution volume, (mg·L^−1^).

(6) $$\ln q_{e}=\ln K_{F}+\frac{1}{n}\ln c_{e}$$

The Langmuir model in linear and non-linear form is expressed by the following relations (see Equations (7) and (8)):

(7) $$\frac{c_{e}}{q_{e}}=\frac{1}{q_{max}K_{L}}+\frac{c_{e}}{q_{max}}$$

(8) $$\frac{1}{q_{e}}=\frac{1}{q_{max}}+\frac{1}{q_{max}K_{L}}+\frac{1}{c_{e}}$$

where:

*q*_max_: the maximum adsorption capacity corresponding to saturation sites, (mg·g^−1^);

*K_L_*: the Langmuir constant, the affinity coefficient between the adsorbent and the adsorbate derived from the affinity constants K (b = 1/K). The remaining parameters have the same meaning as in the equations above.

The Langmuir constant K_L_ is determined empirically and the following relation was used to calculate the separation factor R_L_. See Equation (9) [39]:

(9) $$R_{L}=\frac{1}{1+bc_{i}}$$

where:

*b*: parameter from the equation of the line,

*c_i_*: the initial concentration of metal in solution, (mg·L^−1^),

### 3.5. Methodology of Cd Analysis and Evaluation of Results

The Cd concentration from the aqueous matrix using bottom ash originating from a thermal power plant and biomass combustion was analysed on an AAS contrAA^®^ 700 atomic absorption spectrophotometer with a graphite cuvette (Jena, Germany). The error of determination of the F-AAS method expressed as relative standard deviation (RSD) is 3%.

All the working model solutions of the metals of interest were obtained by successive dilutions of an acidified stock solution of the metal in question with a concentration of 1000 mg·L^−1^. It was prepared by dissolving the appropriate analytical-grade metal salt (CdSO_4_ p.a., Penta), which was dried to constant weight before weighing. The initial concentration and the final concentration of the metal were measured in all cases. Correction for the possible adsorption of metal on the inner surface of the vessel (blind sample) was verified under the same experimental conditions.

## 4. Conclusions

Since the data obtained from the cadmium adsorption study could be described using the pseudo-second-order model, and they also correspond to the linear Langmuir model, several important conclusions can be drawn regarding the mechanism of Cd adsorption and the capacity of the bottom ash.

The pseudo-second-order model suggests that the adsorption process is probably controlled by chemisorption, i.e., the formation of chemical bonds between the adsorbate and the adsorbent surface. Adsorption therefore involves the sharing or transfer of electrons, which is usually a slower process than the physical adsorption. Adsorption is probably strongly influenced by the availability of active sites on the adsorbent surface and the rate at which Cd ions are bound to the surface.

It has been found that the limiting parameter for efficient adsorption of Cd using bottom ash during biomass combustion is its alkaline nature, and it is therefore only applicable to solutions with pH < 2. This is an advantage for the practical application of bottom ash from biomass combustion since the vast majority of industrial wastewater has a pH of 1 up to 2.

It has been proved that 95% of the metal was removed from the solution after 120 min, when the Cd concentration was 100 mg·L^−1^ in aqueous solution and when a sorbent grain-size fraction < 0.5 mm was used. This suggests that the ash from the combustion of plant biomass will have a higher adsorption efficiency, especially for lower cadmium concentrations, which means that it can be used in practice for the treatment of industrial wastewater before discharge to the sewer system.

Based on the thermodynamic balance study, it has been demonstrated that the ash from the combustion of plant biomass can be used for the treatment of acidic industrial wastewater. Cd removal is most effective up to a concentration of 100 mg L^−1^, when approximately 95% of Cd is removed. Above this value, the efficiency of the adsorbent decreases under the given conditions, and at a concentration of 1000 mg L^−1^ Cd, the efficiency of its removal is about 5%. Based on these parameters, the following ashes from the combustion of plant biomass can be recommended for the treatment of acidic industrial waters.

## Figures and Tables

**Table 1:** Chemical composition of the bottom ash sample using the XRF method.

Element	% *w*/*w*	Element	% *w*/*w*
Na	<0.010	Y	0.001
Mg	1.423	Zr	0.022
Al	0.816	Nb	0.0001
Si	28.990	Mo	0.001
P	2.332	Ag	<0.0002
S	2.282	Cd	0.0001
Cl	1.295	Sn	0.0001
K	25.419	Sb	<0.0003
Ca	20.328	Te	<0.0003
Ti	0.131	I	<0.0003
V	0.002	Cs	<0.0004
Cr	0.005	Ba	0.148
Mn	0.125	La	0.011
Fe	1.153	Ce	0.019
Co	<0.0003	Pr	0.005
Ni	0.011	Nd	0.014
Cu	0.007	Hf	0.0001
Zn	0.029	Ta	0.008
Ga	0.001	W	<0.00013
Ge	0.0001	Hg	<0.00007
As	0.0001	Tl	0.0001
Se	0.0001	Pb	0.001
Br	0.004	Bi	<0.0001
Rb	0.018	Th	0.001
Sr	0.087	U	<0.0001

**Table 2:** Phase composition of the tested bottom ash samples.

Phase	Chemical Formula	Reference Card Number PDF-2
Silica	SiO_2_	00-033-1161
Cristobalite	SiO_2_	01-071-6242
Calcite	CaCO_3_	00-005-0586
Tuite	Ca_3_(PO_4_)_3_	00-056-0064
Arcanite	K_2_SO_4_	01-072-0354
Larnite	Ca_2_SiO_4_	00-049-1673
Hematite	Fe_2_O_3_	01-080-5414

**Table 3:** Kinetic parameters of Cd adsorption using bottom ash after combustion of plant biomass for individual grain fractions.

Cadmium								
Grain-Size Fraction mm	q_exp_ mg·g^−1^	Pseudo-First-Order Kinetic Model			Pseudo-Second-Order Kinetic Model			
		q_vyp_ mg·g^−1^	k_1_ min^−1^	R^2^	q_vyp_ mg·g^−1^	k_2_ g·mg^−1^ min^−1^	h_2_ mg g^−1^ min^−1^	R^2^
<0.5	4.64	1.01	80.65	0.871	5.32	0.207	0.81	0.966
0.5–1.0	1.19	2.37	1.159	0.690	1.48	0.129	1.86	0.972
grain not adjusted	1.16	2.50	1.092	0.0002	1.08	0.592	0.55	0.982

**Table 4:** Isothermal model constants and correlation coefficients for cadmium adsorption using bottom ash from plant biomass combustion in a thermal power plant.

**Langmuir Linear**					**Freundlich Linear**		
*q_120_* mg·g^−1^	*q_max_* mg·g^−1^	K_L_ L·mg^−1^	R_L_ pro *c_i_* = 1000 mg·L^−1^	R^2^	K_F_ mg·g^−1^	n	R^2^
2.35	2.38	0.076	0.048	0.992	4.96	7.43	0.651

## Data Availability

Data are contained within the article.
